# Oral Self-Care as a Preventive Strategy in Medicated Older Adults: Biological Mechanisms, Genetic Susceptibility, and Public Health Implications

**DOI:** 10.3390/healthcare14070841

**Published:** 2026-03-25

**Authors:** Nikolaos Koskinas, Mary Gouva, Zoi Konstanti, Eleni Sintou, Stefanos Mantzoukas, Nektaria Zagorianakou

**Affiliations:** 1 Scientific Laboratory for Innovative Technologies in Internal Medicine, Preventive Medicine and Overall Care, Department of Nursing, School of Health Sciences, University of Ioannina, 45500 Ioannina, Greece; n.koskinas@uoi.gr; 2 Scientific Laboratory of Psychology and Person-Centered Care, Department of Nursing, School of Health Sciences, University of Ioannina, 45500 Ioannina, Greece; gouva@uoi.gr (M.G.); smantzoukas@uoi.gr (S.M.); 3 Department of Nursing, School of Health Sciences, University of Ioannina, 45500 Ioannina, Greece; zkon-stanti@uoi.gr; 4 Department of Anesthesia, Patras University Hospital, 26500 Patras, Greece; nelisintou@gmail.com

**Keywords:** polypharmacy, xerostomia, periodontal inflammation, oral microbiome dysbiosis, salivary gland dysfunction, genetic polymorphisms, geriatric oral health

## Abstract

Global population aging has led to a substantial increase in the number of older adults receiving long-term pharmacological treatment, often involving polypharmacy. Long-term medication use is often linked to negative oral health outcomes, such as xerostomia, periodontal disease, dental caries, and changes in the oral microbiome, even if it is necessary for treating systemic conditions. The general health, nutritional state, and quality of life of elderly people are all significantly impacted by these diseases. This narrative review integrates recent data on biological causes, genetic vulnerability, and public health consequences to investigate oral self-care as a preventive strategy in older persons on medication. The effects of long-term medication therapy on oral tissues, salivary function, inflammatory responses, and microbial balance are given special attention, as is the role of genetic variants linked to immunological and inflammatory pathways on individual variability. The review also evaluates oral self-care interventions aimed at reducing medication-related oral complications, such as the use of fluoride, mechanical plaque control measures, and caregiver-assisted oral care practices. Oral self-care is viewed from a public health perspective as a scalable and affordable strategy for reducing oral health disparities in older populations. The results highlight the significance of preventative, individualized, and integrated oral health interventions within larger healthcare frameworks for older persons taking long-term medications.

## 1. Introduction

One of the most significant demographic shifts in the twenty-first century is population aging, which has a profound impact on healthcare systems worldwide. In both industrialized and developing countries, the proportion of older adults is increasing due to extended life expectancy resulting from medical advances, improved living conditions, and public health initiatives [1,2]. The prevalence of chronic non-communicable diseases, such as diabetes, musculoskeletal problems, neurodegenerative diseases, and cardiovascular disorders, rises with longevity. As a result, long-term pharmacological therapy is generally necessary for elderly people, frequently requiring the concurrent use of several drugs [2].

The long-term use of five or more medications is known as polypharmacy, and it has become very frequent among senior citizens. Pharmacotherapy is crucial for managing symptoms and controlling disease, but long-term drug usage is linked to a number of negative consequences that go beyond systemic issues [3,4]. Many commonly prescribed medications, including antihypertensives, antidepressants, anticholinergics, and antidiabetic agents, are associated with adverse oral effects. These include reduced salivary flow, mucosal alterations, and an increased susceptibility to dental caries, periodontal disease, and oral infections [5]. A cumulative detrimental effect on oral and general health may result from these drug-induced oral alterations being made worse by age-related physiological decline, functional limits, and comorbidities [5,6].

Although there is a significant bidirectional relationship between systemic and oral health, oral health is often overlooked in the overall healthcare of older adults. Oral health assessments are infrequently integrated into chronic disease management, and dental care is often excluded from routine medical follow-up [7,8]. Inadequate oral healthcare for older people is caused by a number of obstacles, including limited access to dental treatments, financial limitations, mobility problems, cognitive impairment, and institutionalization. As a result, avoidable oral diseases worsen and have a detrimental impact on social interaction, communication, nutrition, and quality of life. They further heighten the risk of systemic inflammation and related health complications [9,10].

In this regard, dental self-care becomes an essential but little-used preventive measure. Maintaining dental health and halting the course of disease depend heavily on self-care activities, such as regular mechanical plaque removal, proper fluoride use, denture cleanliness, and xerostomia management [11]. Importantly, oral self-care programs are particularly well-suited for older adults receiving long-term pharmacotherapy, as they can be tailored to individual functional capacities and supported by caregivers when necessary. Aligned with contemporary models of personalized and preventive medicine, oral self-care promotion offers a scalable, sustainable, and cost-effective strategy to reduce oral disease burden [12,13]. While previous reviews have addressed geriatric oral health or medication-related oral complications separately, fewer studies have examined these issues through an integrated framework that combines biological mechanisms, genetic susceptibility, and public health perspectives. This narrative review examines oral self-care among medicated older adults by analyzing biological mechanisms, genetic susceptibility, and public health implications, and highlights its importance in integrated healthcare for aging populations.

## 2. Methodology

This narrative review aims to synthesize current evidence on oral self-care as a preventive strategy in pharmacologically treated older adults, with particular emphasis on biological mechanisms, genetic susceptibility, and public health implications. The methodological approach follows commonly accepted principles for narrative reviews, which aim to provide a comprehensive and integrative overview of a topic by synthesizing evidence from diverse study designs and sources [14,15].

A structured literature search was conducted using the electronic databases PubMed, Web of Science, and Scopus. The search included combinations of keywords such as oral self-care, older adults, aging, polypharmacy, xerostomia, periodontal disease, oral microbiome, genetic susceptibility, and public health. Reference lists of key publications were also manually screened to identify additional relevant studies.

For the purposes of this review, primary sources were defined as original research studies reporting empirical data, including randomized controlled trials, observational studies, cohort studies, and clinical investigations related to oral health in older adults. Secondary sources included systematic reviews, narrative reviews, and clinical practice guidelines that synthesize existing evidence and provide broader clinical or public health perspectives.

Because this article follows a narrative review design, evidence from both primary research and high-level secondary sources was considered in order to provide a comprehensive overview of the biological, clinical, and preventive dimensions of oral health in medicated older adults.

The search focused primarily on publications from the last 20 years, a period that reflects substantial advances in geriatric medicine, pharmacotherapy, oral microbiome research, and preventive oral health strategies. Earlier foundational studies were included when they were considered essential for understanding key biological mechanisms or conceptual frameworks.

The retrieved literature was screened for relevance to the objectives of the review. Studies addressing medication-related oral effects, preventive oral self-care strategies, biological mechanisms, and public health aspects of geriatric oral health were prioritized (Table 1). The selected evidence was then qualitatively synthesized, with emphasis on recurring themes and mechanistic insights relevant to preventive oral healthcare in aging populations.

## 3. Oral Health Challenges in Elderly Individuals

The oral cavity undergoes several physiological changes associated with aging, increasing susceptibility to a range of oral health conditions among older adults [16]. Increased susceptibility to injury, inflammation, and infection results from structural and functional changes in oral tissues, including thinning of the oral mucosa, reduced regenerative capacity, and alterations in periodontal support [17,18]. Furthermore, the host’s ability to respond to microbial assaults may be compromised by age-related changes in immune function and inflammatory control, which could hasten the development of oral conditions [19].

The decrease in salivary flow is one of the most common and clinically important age-related alterations. Salivary gland hypofunction is common among older adults, particularly those receiving long-term pharmacological treatment, although it is not an inevitable consequence of aging. Reduced salivary flow compromises oral homeostasis and increases the risk of dental caries, mucosal irritation, oral infections, and difficulties with speaking and eating [20,21]. These defense mechanisms are compromised by decreased salivary secretion, which raises the risk of dental cavities, mucosal irritation, oral infections, and problems speaking and eating.

Elderly people’s declining oral health is made worse by cognitive decline and motor limitations. Effective oral hygiene activities, such as toothbrushing and interdental cleaning, may be impeded by conditions including dementia, Parkinson’s disease, and stroke [22]. Daily oral self-care may be compromised by reduced manual dexterity, visual impairment, and musculoskeletal limitations, even among individuals who are cognitively intact. These functional barriers are associated with increased plaque accumulation and the underrecognition of oral health conditions [23].

Dependency and institutionalization are two more risk factors for poor oral health outcomes. In long-term care settings, poor oral hygiene among older adults is commonly attributed to inadequate caregiver training, time constraints, and the low prioritization of oral care. Reliance on caregivers for activities of daily living may lead to inconsistent or inadequate oral hygiene practices, thereby increasing the prevalence of untreated oral conditions [24,25].

Elderly populations have a high prevalence of oral illnesses due to the combined influence of these variables. Gingival recession, decreased salivary flow, and increased root surface exposure all contribute to the high prevalence of dental caries, especially root caries [26]. Age-related immunological changes and cumulative lifetime exposure to risk factors both contribute to periodontal disease, which continues to be a major cause of tooth loss in older persons. Xerostomia represents one of the most frequently reported oral symptoms in older adults and is significantly associated with impaired quality of life, independent of its underlying etiology [27]. Denture use, reduced salivary flow, and systemic comorbidities are frequently associated with oral infections, particularly those caused by *Candida* species. Beyond compromising oral function, tooth loss and prosthesis-related complications, including poor denture fit, mucosal irritation, and inadequate hygiene, are associated with an increased risk of infection and inflammation [28].

These oral health conditions highlight the interplay among biological aging, functional limitations, and environmental factors affecting older adults. Developing successful preventive strategies, especially those focused on oral self-care and caregiver-supported treatments catered to the needs of elderly people, requires an understanding of these complex variables.

## 4. Impact of Chronic Drug Treatment on Oral Health

### 4.1. Polypharmacy and Oral Adverse Effects

Chronic pharmacological treatment is a defining feature of healthcare in older adults and plays a significant role in shaping oral health outcomes in this population [29]. The high frequency of polypharmacy, which frequently entails the long-term use of drugs from several therapeutic groups, is a reflection of the cumulative burden of chronic diseases. Although necessary for disease management, polypharmacy increases the risk of adverse drug reactions, including those affecting the oral cavity [30]. Oral side effects may be exacerbated by medication-drug interactions, extended exposure, and age-related changes in drug metabolism, especially in frail elderly people with numerous comorbidities [6].

Since oral health evaluations are rarely included in normal medical follow-up, oral problems linked to polypharmacy are often underrecognized in clinical practice. This oversight could lead to the spread of avoidable oral diseases and a delayed diagnosis of medication-related oral symptoms [5].

### 4.2. Drug-Induced Xerostomia and Salivary Gland Dysfunction

Salivary gland hypofunction, which results in xerostomia, is one of the most frequent and clinically important oral adverse effects of long-term medication use [31]. Antihypertensives, antidepressants, antipsychotics, anticholinergics, and diuretics are among the many drugs that are frequently provided to elderly patients and interfere with the autonomic regulation of salivary production. Reduced salivary flow compromises the essential protective functions of saliva, including lubrication, buffering capacity, antimicrobial activity, and the remineralization of dental hard tissues [32,33].

Xerostomia is linked to a lower quality of life, changed taste perception, mucosal pain, poor swallowing and mastication, and an increased risk of dental cavities in older persons on medication. Due to variations in drug schedules, biological sensitivity, and oral self-care habits, the severity of xerostomia-related problems varies significantly among individuals [34,35].

### 4.3. Periodontal and Mucosal Alterations Associated with Medications

Chronic medication therapy can cause a variety of periodontal and oral mucosal abnormalities in addition to salivary dysfunction [16]. Gingival overgrowth has been associated with several classes of medications, including calcium channel blockers, immunosuppressive agents, and anticonvulsants, which may impair plaque clearance and exacerbate inflammatory periodontal disease. Other drug classes may cause oral ulcers and dysesthesia, worsen wound healing, or increase mucosal fragility [36,37].

Particularly in elderly persons with weakened immune systems or poor oral hygiene, these drug-related changes may make pre-existing periodontal disease worse. If medication-related factors are not adequately addressed, plaque accumulation and inflammation may persist despite conventional treatment, underscoring the need for targeted preventive measures [38].

### 4.4. Medications Affecting Bone Metabolism and Oral Tissues

Long-term exposure to medications influencing bone metabolism, including bisphosphonates and other antiresorptive or antiangiogenic agents, further complicates oral health management in older adults [39,40]. These medications have been linked to medication-related osteonecrosis of the jaw, an uncommon but dangerous illness that is frequently brought on by invasive dental treatments or persistent oral infections. Although the absolute risk remains low, the potential severity of this outcome underscores the importance of early intervention and preventive oral care [41].

For older patients undergoing these treatments, thorough oral health evaluation and management are therefore essential. As summarized in Table 2, several drug classes frequently prescribed in elderly populations are associated with a range of oral adverse effects. In addition to reducing the risk of serious medication-related complications, preventive oral self-care combined with routine professional monitoring may decrease the need for invasive dental procedures.

## 5. Oral Self-Care as a Preventive and Interventional Tool

### 5.1. Mechanical Plaque Control and Fluoride-Based Prevention

The cornerstone of oral self-care is mechanical plaque management, which is crucial for preventing periodontal disease and tooth caries in older persons. Effective plaque control is essential in medicated older adults due to increased inflammatory susceptibility, reduced salivary protection, and cumulative oral risk exposure [51]. Regular toothbrushing with fluoride toothpaste remains the most practical and accessible preventive strategy, while interdental cleaning further reduces plaque accumulation in areas vulnerable to periodontal deterioration [52].

The efficacy of traditional oral hygiene procedures may be jeopardized by age-related functional decline, which includes diminished manual dexterity and visual impairment [53]. Plaque control and adherence can be enhanced by adaptive techniques, including powered toothbrushes, altered handles, and streamlined procedures. High-fluoride toothpaste and topical fluoride treatments are examples of fluoride-based preventative strategies that are essential for improving enamel and root surface resistance to demineralization, especially in people with gingival recession and xerostomia [54].

### 5.2. Management of Xerostomia and Salivary Dysfunction

Xerostomia is a frequent complication in older adults receiving long-term pharmacological treatment and represents an important target for preventive oral self-care. Management strategies include adequate hydration, saliva substitutes, and the use of non-cariogenic chewing gum or lozenges to stimulate residual salivary function [20,55].

To reduce additional mucosal irritation and the risk of caries, it is also advised to avoid mouthwashes that contain alcohol and acidic or sugary items. Adjunctive antimicrobial or remineralizing agents may be selectively employed to support oral health in high-risk individuals [56,57]. Since symptom severity and clinical significance vary greatly among senior patients, it is crucial to customize xerostomia therapy procedures to individual needs and medication profiles [5].

### 5.3. Denture Hygiene and Prosthesis Care

For older persons who have lost all or part of their teeth, denture maintenance and hygiene are essential components of oral self-care. Inadequate cleaning of a detachable prosthesis raises the risk of mucosal damage, *Candida* infections, and denture-related stomatitis by encouraging biofilm formation. Essential preventive measures include routine removal of dentures during sleep, daily mechanical cleaning, and appropriate storage practices [58].

Adherence to appropriate denture hygiene practices may be compromised by age-related cognitive decline, physical dependency, and institutional life. These circumstances frequently necessitate caregiver involvement to maintain continuity of care. Poorly fitting or damaged prostheses exacerbate mucosal inflammation and functional impairment, underscoring the importance of routine professional evaluation in addition to effective self-care practices [59].

In addition to hygiene practices, oral self-care may also contribute to the early detection of oral pathology. Older adults represent a population at increased risk for oral potentially malignant disorders and oral cancer. Encouraging periodic oral self-examination may facilitate earlier identification of suspicious lesions, such as persistent ulcers, mucosal discolorations, or unexplained tissue changes. When combined with regular professional examinations, awareness of early warning signs may improve timely referral and diagnosis, thereby enhancing clinical outcomes.

### 5.4. Educational and Caregiver-Assisted Interventions

Education, motivation, and outside assistance all have a significant impact on the efficacy of oral self-care. Interventions targeting oral health literacy in older adults have been shown to positively influence clinical outcomes and self-care behaviors. However, self-directed care might not be enough for people with cognitive impairment or functional limitations [60,61].

In dependent and institutionalized elderly populations, caregiver-assisted dental care is a crucial therapeutic technique. Training caregivers to manage medication-related oral conditions, recognize early signs of oral disease, and perform daily oral hygiene tasks can significantly improve oral health outcomes. Compared to non-interventional methods, structured, supervised oral care programs are typically more successful, highlighting the significance of ongoing reinforcement and active support [12].

Although several interventions promoting oral self-care in older adults have demonstrated positive effects on oral hygiene behaviors and clinical outcomes, the available evidence remains heterogeneous. Previous reviews have noted substantial variation in study design, intervention characteristics, and outcome measures, which limits direct comparability and the strength of conclusions. Furthermore, functional and cognitive limitations in frail older adults may reduce the effectiveness of self-directed interventions. As highlighted in a scoping review by Gomez-Rossi et al., the current body of evidence regarding interventions to improve oral health in older populations remains fragmented and insufficiently comparable to draw robust conclusions [62].

### 5.5. Oral Self-Care as a Bridge Between Individual and System-Level Prevention

Oral self-care serves as a bridge between clinical practice and public health prevention, extending beyond individual behavioral practices. Self-care measures can lessen the negative effects of long-term medication therapy on oral health and the need for complicated dental procedures when they are accompanied by education, caregiver involvement, and healthcare integration [63]. Therefore, within larger healthcare frameworks for aging populations, oral self-care is a useful, flexible, and economical preventive technique.

Oral self-care may also contribute to the early recognition of oral pathological changes. Older adults are at increased risk for oral potentially malignant disorders and oral cancer due to cumulative exposure to risk factors and age-related biological changes. Encouraging awareness of persistent ulcers, mucosal discoloration, or unexplained lesions may support earlier clinical evaluation and diagnosis, thereby improving clinical outcomes [64].

Key oral self-care interventions, their preventive targets, and specific considerations for medicated older adults are summarized in Table 3.

## 6. Genetic and Biological Susceptibility in Oral Health Among Medicated Older Adults

### 6.1. Genetic Polymorphisms and Inflammatory Response

Individual responses to microbial challenge and inflammatory stimuli in the oral cavity are partly determined by genetic susceptibility [72]. Polymorphisms in genes involved in immune and inflammatory regulation, including interleukin-1, interleukin-6, and tumor necrosis factor-α, have been linked to both the risk and severity of periodontal disease. The impact of these genetic variations may be amplified in older persons due to age-related immunological dysregulation, leading to increased inflammatory responses and faster periodontal tissue degradation [73,74].

Genetic predisposition may further influence oral health outcomes in the setting of long-term pharmaceutical treatment. Medications that influence inflammatory signaling or immune function may interact with host genetic factors to exacerbate periodontal inflammation or impair tissue healing [75]. These interactions may partially explain the substantial variability in oral disease presentation observed among medicated older adults exposed to similar environmental and behavioral risk factors [76].

### 6.2. Salivary Function and Biological Biomarkers in Oral Disease Susceptibility

Variation in oral disease susceptibility is influenced not only by genetic differences but also by biological variability in salivary composition and function. Saliva contains several protective components, including immunoglobulins, antimicrobial peptides, enzymes, and buffering agents that contribute to oral homeostasis. Oral health outcomes in older persons may be greatly impacted by variations in saliva quantity and quality, which are influenced by systemic, pharmaceutical, and genetic factors [77,78].

The use of salivary biomarkers as non-invasive measures of dental and systemic health has grown. Changes in pH, inflammatory mediators, and salivary flow rate could be indicators of underlying biological susceptibility and disease activity. These biomarkers may assist in identifying people who are more susceptible to inflammatory oral disorders and complications connected to xerostomia, as well as shed light on the effects of long-term drug use on oral tissues in older people on medication [79,80,81].

### 6.3. Implications for Personalized Preventive Strategies

Understanding genetic and biological predisposition is important for the development of individualized preventive oral healthcare [82]. Preventive decision-making may be guided by knowledge of individual risk profiles based on clinical history, pharmaceutical exposure, and biological indicators, even though comprehensive genetic testing is currently not practical in routine clinical practice [83]. Increased oral self-care practices, more regular professional monitoring, and focused preventative therapies may be beneficial for older persons with elevated inflammatory responses or less salivary protective capability [51].

Personalized oral self-care strategies that emphasize early intervention and risk-based care align with contemporary principles of preventive medicine. Oral healthcare professionals may maximize self-care recommendations and enhance outcomes for senior patients receiving long-term pharmaceutical treatment by including genetic and biological factors into preventive methods [82]. This viewpoint supports oral self-care as an adaptable and flexible preventative strategy that can address interindividual variation in the risk of oral illness. Table 4 summarizes the main biological and genetic factors that affect older persons using medication’s susceptibility to oral illness and their preventative implications.

## 7. Oral Microbiome, Inflammation, and Systemic Health

### 7.1. Aging, Medication Use, and Oral Microbiome Dysbiosis

Maintaining oral and systemic equilibrium depends heavily on the diverse and dynamic microbiota of the oral cavity. Chronic illness, long-term medication use, and age-related physiological changes in older persons can upset the microbial balance and encourage dysbiosis [93]. Pharmaceutical-induced reductions in salivary flow, impaired immune function, and alterations in oral pH create an environment conducive to the proliferation of cariogenic and periodontopathogenic microorganisms [94].

These microbiome changes in elderly people on medication are frequently exacerbated by functional limits and poor oral hygiene, hastening the onset of mucosal infections, periodontal disease, and dental caries. Both xerostomia and chronic inflammatory conditions are associated with reduced microbial diversity and increased pathogenic load, highlighting the vulnerability of older adults receiving long-term medication. These microbial alterations may also contribute to persistent oral inflammation and its systemic consequences [95].

### 7.2. Oral Inflammation and Systemic Disease Associations

Chronic oral inflammation, particularly within periodontal tissues, is closely associated with dysbiosis of the oral microbiota and may contribute to systemic inflammatory burden [96]. In older adults, periodontal inflammation has been linked to several systemic conditions, including frailty, respiratory infections, diabetes mellitus, and cardiovascular disease [8]. Given that systemic inflammation plays a key role in disease progression and functional decline in aging populations, the bidirectional relationship between periodontal disease and systemic health is of particular importance. These associations highlight the need to consider oral health as an integral component of comprehensive healthcare for older adults [97].

### 7.3. Role of Oral Self-Care in Modulating Microbial and Inflammatory Burden

Oral self-care plays a central role in maintaining microbial balance and limiting inflammatory processes in the oral cavity [98]. For breaking up pathogenic biofilms and reestablishing microbial equilibrium, mechanical plaque removal remains the most effective strategy [99]. Maintenance of oral homeostasis and mucosal integrity may be enhanced through adjunctive preventive interventions, including fluoride-based therapies, targeted antimicrobial agents, and effective denture hygiene practices [100].

Particularly in medicated older persons with enhanced biological sensitivity, consistent and supportive self-care activities may attenuate microbiome dysbiosis and minimize chronic inflammation [101]. Oral self-care may also indirectly improve systemic health outcomes by reducing oral bacterial load and inflammatory activity. This finding supports the role of oral self-care as a clinically relevant and biologically plausible preventive component within integrated healthcare models for aging populations [95].

## 8. Public Health Perspective

From the standpoint of public health, oral health in older persons is an increasingly important yet underappreciated issue [27]. Although oral diseases are highly prevalent among older adults, oral health continues to receive limited attention within public health agendas, especially in relation to chronic disease management and aging policies. Because oral difficulties associated with polypharmacy and systemic sickness disproportionately impact older persons receiving long-term pharmaceutical treatment, this negligence is particularly troublesome for them [102].

Elderly people have significant disparities in oral health, which are impacted by living conditions, educational attainment, socioeconomic status, and access to healthcare services [103]. Access to appropriate dental care is particularly challenging for older adults residing in long-term care facilities, those with limited financial resources, or individuals with restricted mobility [104]. Dental treatments are not properly integrated into publicly funded care in many healthcare systems, which leads to treatment delays and avoidable disease progression. These differences underscore the necessity of preventive measures that are easily accessible, reasonably priced, and flexible enough to fit a variety of living situations [105].

The low cost, scalability, and potential for widespread implementation of oral self-care make it a very valuable public health intervention. Preventive self-care practices can be promoted through community-based programs, primary healthcare settings, and long-term care facilities to reduce reliance on specialist dental services [106]. Educational interventions targeting older adults, caregivers, and healthcare professionals have led to improvements in oral hygiene behaviors and oral health outcomes. Integrating oral self-care into broader health promotion programs may enhance adherence and reinforce the importance of oral health as a component of overall well-being [107].

Within public health settings, elderly populations who are institutionalized and dependent need specific consideration. Due to personnel shortages, inadequate training, and conflicting care demands, dental care is often deprioritized in these settings. To ensure consistent preventative care, it is crucial to have standardized oral care protocols that are backed by institutional policy and caregiver education. Public health initiatives that recognize caregiver-assisted oral care as an integral component of routine healthcare may substantially reduce the burden of oral disease in long-term care settings [104,108].

One of the most important steps toward comprehensive preventive care is the integration of oral health into primary and geriatric healthcare services. Early detection of medication-related oral hazards and prompt preventative measures can be facilitated by interdisciplinary collaboration between dental experts and other healthcare providers [109]. Acknowledging oral self-care as a public health priority at the policy level may facilitate the development of guidelines, training programs, and reimbursement models aimed at improving oral health outcomes among older populations [110].

Financing preventive oral health interventions for older adults represents an important challenge in many healthcare systems. In several countries, dental services remain only partially covered by public health programs, which may limit access to preventive care for older populations. Integrating oral health promotion and preventive self-care strategies into primary healthcare services, community health programs, and insurance reimbursement frameworks could improve accessibility and long-term sustainability [109,110]. Although financing models vary across countries, strengthening preventive coverage and incorporating oral health within broader geriatric care policies may help reduce the long-term burden of medication-related oral diseases in aging populations [105].

Strengthening public health strategies that promote oral self-care may reduce oral health disparities. Healthcare systems can transition to more inclusive and preventive modes of treatment for medicated older individuals by defining oral self-care as both a personal obligation and a public health initiative [111].

## 9. Future Directions and Preventive Strategies

### 9.1. Personalized and Risk-Based Preventive Approaches

Personalized and risk-based methods should become more prevalent in future oral healthcare preventive measures for older persons on medication. Advances in understanding genetic susceptibility, inflammatory profiles, and oral microbiota dynamics have enabled the identification of individuals at increased risk of oral disease [112]. Combining clinical signs, drug profiles, and biological markers may enable more specialized preventive interventions, even though systematic genetic screening is currently impractical in clinical practice. In order to reduce medication-related oral problems, high-risk patients may benefit from more stringent oral self-care practices, closer professional supervision, and focused preventive efforts [113].

### 9.2. Technological and Interdisciplinary Innovations

New options to enhance preventative oral healthcare in older populations are made possible by technological breakthroughs. Tele-dentistry, mobile health applications, and remote monitoring systems may enhance access to preventive care, particularly for homebound or institutionalized older adults. These tools can facilitate early identification of oral health problems, promote self-care practices, and enhance communication among patients, caregivers, and healthcare providers [114,115].

The effective application of preventive measures requires interdisciplinary cooperation. Recognizing medication-related oral hazards may be enhanced by incorporating oral health evaluations into primary and geriatric care and involving doctors, nurses, pharmacists, and caregivers. Given their role in medication management, pharmacists are strategically positioned to identify xerogenic agents and deliver preventive counseling [116].

### 9.3. Research and Policy Priorities

Future research should focus on evaluating the effectiveness of comprehensive preventive programs that integrate technological support, caregiver involvement, and oral self-care education [117]. To evaluate the effect of preventative measures on oral and systemic health outcomes in senior populations using medication, longitudinal studies are required. It may be easier to design recommendations, training programs, and reimbursement models that support preventive treatment at the policy level if oral health is given priority within frameworks related to aging and chronic diseases [118,119,120].

## 10. Conclusions

A complicated interplay between biological aging, medication-related side effects, functional constraints, and social determinants of health affects oral health in older persons undergoing long-term pharmaceutical treatment. Preventable oral diseases, such as dental caries, periodontal disease, xerostomia, and oral infections, are predicted to put an increasing strain on people and healthcare systems as the population ages and polypharmacy increases. These conditions are still largely preventable with targeted preventive measures, despite their high prevalence and negative effects on overall health and quality of life.

This narrative review highlights oral self-care as a crucial, cost-effective, and sustainable preventive strategy for mitigating the oral effects of aging and long-term pharmacological use. Effective oral self-care can significantly reduce disease risk and progression when supported by patient education, caregiver involvement, professional guidance, and individualized risk assessment. These findings are consistent with previous reviews that emphasize the impact of polypharmacy and medication-related oral complications in aging populations. However, the present review expands the existing literature by integrating biological mechanisms, genetic susceptibility, and public health perspectives with practical oral self-care strategies for medicated older adults [9,29,102].

Reducing oral health disparities requires addressing access barriers, improving caregiver education, and incorporating oral health promotion into primary and geriatric healthcare services. Prioritizing oral self-care within interdisciplinary healthcare frameworks is consistent with preventive medicine concepts and may promote healthy aging while lowering the burden of preventable oral illness in older persons.

## Figures and Tables

**Table 1:** Summary of the literature search strategy used in this narrative review.

Component	Description
Databases searched	PubMed, Web of Science, Scopus
Keywords	“oral self-care”, “older adults”, “aging”, “polypharmacy”, “xerostomia”, “periodontal disease”, “oral microbiome”, “genetic susceptibility”, “public health”
Search approach	Keywords combined using Boolean operators (AND/OR)
Types of sources	Original research articles, randomized controlled trials, observational studies, systematic reviews, narrative reviews, and clinical practice guidelines
Time frame	Primarily studies published within the last 20 years
Language	English

**Table 2:** This table summarizes commonly reported oral adverse effects of drug classes frequently prescribed in elderly populations. Individual risk varies depending on dosage, duration of treatment, comorbidities, and oral self-care practices.

Drug Class	Common Examples	Primary Oral Adverse Effects	Underlying Mechanisms	Preventive and Self-Care Considerations
Antihypertensives [42]	Beta-blockers, ACE inhibitors, calcium channel blockers	Xerostomia, gingival overgrowth, taste alteration	Autonomic interference; altered vascular permeability	Enhanced plaque control; xerostomia management; regular periodontal monitoring
Antidepressants and antipsychotics [43,44]	SSRIs, TCAs, antipsychotics	Xerostomia, dysphagia, increased caries risk	Anticholinergic effects; reduced salivary secretion	Saliva substitutes; fluoride use; dietary counseling
Anticholinergics [32]	Antispasmodics, antiparkinsonian agents	Severe xerostomia, mucosal discomfort	Inhibition of parasympathetic salivary stimulation	Frequent hydration; saliva stimulants; avoidance of alcohol-based products
Diuretics [45]	Thiazides, loop diuretics	Xerostomia, altered taste perception	Fluid depletion; electrolyte imbalance	Hydration strategies; remineralizing agents
Calcium channel blockers [46,47]	Nifedipine, amlodipine	Gingival overgrowth, periodontal inflammation	Fibroblast stimulation; collagen accumulation	Intensive plaque control; professional periodontal care
Anticonvulsants [48]	Phenytoin	Gingival enlargement, mucosal changes	Altered fibroblast activity	Rigorous oral hygiene; caregiver-assisted care
Immunosuppressants [49]	Cyclosporine	Gingival overgrowth, opportunistic infections	Immune modulation; tissue response alteration	Infection prevention; mucosal surveillance
Antiresorptive and antiangiogenic agents [50]	Bisphosphonates, denosumab	Osteonecrosis of the jaw (rare), delayed healing	Inhibition of bone remodeling; vascular changes	Preventive dental care; avoidance of invasive procedures; close monitoring

**Table 3:** This table summarizes key oral self-care interventions and their preventive targets in older adults receiving chronic pharmacological treatment, with emphasis on mechanisms of action and age-related considerations.

Self-Care Domain	Specific Interventions	Primary Preventive Targets	Mechanism of Action	Special Considerations in Elderly Populations
Mechanical plaque control [65]	Toothbrushing with fluoridated toothpaste; interdental cleaning	Dental caries; periodontal disease	Biofilm disruption; reduction in inflammatory burden	Use of powered toothbrushes; adapted handles; simplified routines for reduced dexterity
Fluoride-based prevention [66]	Standard and high-fluoride toothpaste; topical fluoride applications	Root caries; enamel demineralization	Enhancement of remineralization; increased resistance to acid challenge	Particularly beneficial in xerostomia and gingival recession
Xerostomia management [67]	Hydration; saliva substitutes; saliva stimulants	Caries; mucosal inflammation; oral discomfort	Restoration of lubrication; partial compensation for salivary loss	Tailored to medication profile and symptom severity
Denture hygiene and prosthesis care [68,69]	Daily mechanical cleaning; overnight removal; proper storage	Denture stomatitis; Candida infections; mucosal trauma	Biofilm reduction; mucosal recovery	Caregiver assistance often required for dependent individuals
Educational interventions [70]	Oral health education; motivational reinforcement	Poor adherence; plaque accumulation	Behavior modification; improved self-care compliance	Adapted to cognitive status and health literacy
Caregiver-assisted oral care [13]	Supervised brushing; prosthesis care; symptom monitoring	Advanced oral disease; neglect-related complications	Ensures continuity of care; early disease detection	Essential for institutionalized and cognitively impaired patients
Integrated preventive approach [71]	Combined self-care, caregiver support, and professional monitoring	Progression of medication-related oral disease	Risk reduction; early intervention	Bridges individual care and healthcare systems

**Table 4:** This table highlights major genetic and biological factors influencing oral disease susceptibility in older adults under chronic medication and their implications for personalized preventive oral care.

Factor	Associated Oral Condition(s)	Underlying Biological Mechanism	Interaction with Aging and Medication	Implications for Preventive Oral Self-Care
Pro-inflammatory cytokine polymorphisms (e.g., IL-1, IL-6, TNF-α) [84,85]	Periodontal disease; tissue breakdown	Enhanced inflammatory response to microbial biofilms	Amplified by immune senescence and immunomodulatory drugs	Intensified plaque control; closer periodontal monitoring
Genetic variability in immune regulation [86]	Periodontal disease; oral infections	Altered host–microbe interaction	Increased susceptibility in polypharmacy and chronic disease	Early preventive intervention; caregiver support where needed
Reduced salivary flow and altered salivary composition [87,88]	Dental caries; xerostomia; mucosal discomfort	Decreased antimicrobial activity and buffering capacity	Exacerbated by xerogenic medications	Xerostomia management; fluoride-based prevention
Altered salivary biomarkers (pH, inflammatory mediators) [89,90]	Caries progression; mucosal inflammation	Reflection of local and systemic inflammatory status	Influenced by chronic drug exposure	Risk stratification; tailored self-care protocols
Age-related epigenetic and tissue repair changes [91,92]	Delayed healing; chronic inflammation	Reduced regenerative capacity	Compounded by long-term pharmacotherapy	Emphasis on prevention and avoidance of invasive procedures

## Data Availability

No new data were created or analyzed in this study.
